# Examining the burden of unintentional injuries in Ghana: A systematic review and meta-analysis

**DOI:** 10.1016/j.afjem.2025.100907

**Published:** 2025-09-18

**Authors:** Daniel Gyaase, Deepti Beri, Stacie Powell, Nipuna Cooray, Margie Peden, Julie Brown, Jagnoor Jagnoor

**Affiliations:** a Injury Division, The George Institute for Global Health, Faculty of Medicine and Health, University of New South Wales, Sydney, Australia; b Department of Epidemiology and Biostatistics, School of Public Health, Kwame Nkrumah University of Science and Technology, Kumasi, Ghana; c Injury Division, The George Institute for Global Health, New Delhi, India; d Global Injury Program, The George Institute for Global Health, UK; e School of Public Health, Imperial College London, UK

**Keywords:** Unintentional injuries, Road traffic injury, Drowning, Burns, Poisoning, Falls, Burden

## Abstract

**Background:**

In Ghana, there is a lack of comprehensive and empirical data on injuries. In the absence of robust national datasets, systematic reviews serve as a critical tool for understanding existing evidence. Our study synthesises the available literature to estimate the pooled prevalence and mortality associated with unintentional injuries in Ghana.

**Method:**

We searched and identified studies that reported on the burden (prevalence, mortality, economic and disabilities) of commonly reported unintentional injuries (road traffic injuries, falls, burns, drowning and poisoning) in Ghana. Studies were identified from PubMed, EMBASE, Global Health, and Scopus from 2000 to 2023.

**Result:**

A total of 46 studies were included in the review. The prevalence and mortality of unintentional injuries were high, with a pooled estimate of 18 % (95 % CI: 11 % – 26 %) and 15 % (9 % CI: 10 % – 21 %), respectively. Road traffic injury (RTI) was found to be a major contributor to the high prevalence and mortality. Our review found limited data on the economic burden and disabilities from unintentional injuries. Despite the lack of complete data, the cost of treating unintentional injuries appears to be significantly high. The annual cost of treating RTIs was US$6730,862.89, falls were US$1645,736.50, and burns were US$464,937.11.

**Conclusion:**

Our review found a high prevalence, mortality, and likely economic burden of unintentional injuries in Ghana. Prioritising road safety could significantly reduce the burden of unintentional injuries in Ghana. Due to the limited studies on the economic burden and disability from unintentional injuries, more research is needed to drive insurance policies and rehabilitation practices.

**African relevance**

• In Ghana and other African countries, there is significant under-reporting of injury (such as road injuries, drowning, etc.) in national databases, and a review such as this helps to understand the burden and plan for mitigation strategies and policies
• The African region is noted as the global capital for road fatalities, aligning with the high prevalence and mortality of road traffic injuries in this review.
• The findings call for swift attention and prioritisation of injury prevention in public health agendas in Ghana and on the African continent
• The review calls for more research on the economic burden and disability associated with unintentional injuries in Ghana and the broader African context

## Background

Injuries account for 8 % of all deaths globally, of which 72 % result from unintentional injuries [1]. Deaths from injuries are 1.7 times higher than deaths from diseases such as HIV/AIDs, malaria, and tuberculosis globally [2,3]. Fourteen per cent of all Disability Adjusted Life Years (DALYs) are attributed to injuries, of which 90 % are from low- and middle-income countries (LMICs) [4]. The majority of deaths from road traffic injuries (RTIs) (93 %) [5], falls (80 %) [6], unintentional drownings (90 %) [7], and burns (90 %) [8] occur in LMICs [1].

In sub-Saharan Africa, including Ghana, the burden of unintentional injuries is rising exponentially [9]. However, the true burden of injury remains unclear, as sub-Saharan African countries, lack comprehensive and reliable datasets, as previously reported [10,11]. This is also true for Ghana, where there is no nationwide injury registry or surveillance system. Existing national data sources are limited to hospital records and the police crash database. However, limited formal access to healthcare hinders the utility of hospital data as a comprehensive source for injury surveillance [12]. The police crash database, which focuses solely on RTIs, is further constrained by significant under-reporting [11,13].

A potential alternative source of information on injuries is the literature, where individual research studies have comprehensively reported data on injuries, including those beyond RTI, such as drowning, falls, burns, and poisoning. In the absence of comprehensive national data sources, systematically collating data from literature may provide potential insights into the relative burden of different types of unintentional injury. Yet, to our knowledge, no such assessment of existing literature on the burden of unintentional injuries has been conducted in Ghana. This study aims to systematically review the available literature and synthesise empirical evidence on the burden (prevalence, mortality, economic and disability) of commonly reported unintentional injuries (RTIs, falls, drowning, burns, and poisoning) in Ghana.

## Methods

The systematic review was reported in accordance with the Preferred Reporting Items for Systematic Review and Meta-Analyses (PRISMA) 2020 checklist [14]. The condition, context, and population (CoCoPop) [15] criterion was used to frame our search. For this review, the condition was unintentional injuries (RTIs, falls, drowning, burns, and poisoning), the context is Ghana (national and subnational), and the population consisted of the entire population of the country (individuals who had suffered any of the types of injuries specified or not). The protocol for the review was registered with PROSPERO with registration number CRD42023414491.

### Inclusion criteria

Studies were included if they met the following criteria:

• The study reported on any of the following unintentional injuries: RTIs, falls, drowning, burns and unintentional poisoning.
• Studies conducted in Ghana (national and subnational) from 2000 to 2023.
• All types of quantitative study designs
• All age groups.
• The study reported data on either prevalence/proportion/frequency, mortality, disability, or cost (economic burden) of any of the stated unintentional injuries.

*Exclusion criteria*: We excluded studies conducted before 2000, qualitative studies, and literature/systematic reviews,

### Information sources and search strategy

We searched four electronic databases (PubMed, EMBASE, Global Health, and Scopus) for original research studies published between 2000 and 2023. DG conducted a database search in consultation with a librarian at the University of New South Wales. The search was conducted on November 5, 2022, and updated on July 7, 2024. The search strategy was developed to include several Medical Subject Headings (MeSH) terms and keywords relevant to unintentional injuries and Ghana. The search identified all published studies on RTIs, falls, drowning, burns, and unintentional poisoning in Ghana.

### Selection of studies

**Fig. 1:**
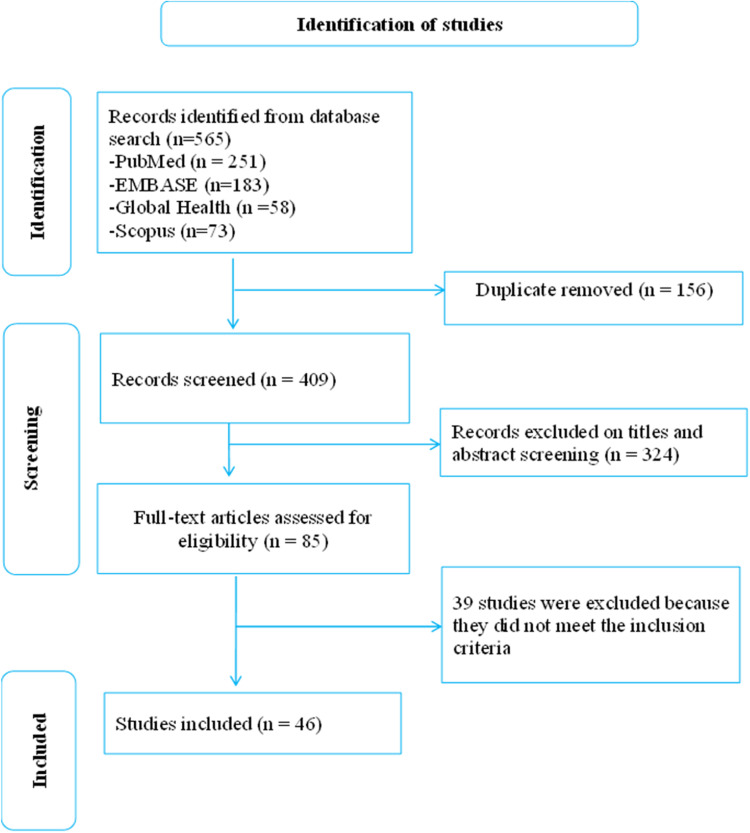
PRISMA Flow diagram of included studies.

We uploaded all identified studies into EndNote 20 reference manager and removed duplicates. After duplicate removal, we exported the studies to Rayyan, a cloud-based artificial intelligence-guided screening platform. Two reviewers (DG and DB) independently screened the articles based on titles and abstracts and included or excluded studies based on the inclusion criteria. Disagreements were resolved by discussion between the two reviewers. In the second phase, we retrieved the full texts of studies that both reviewers marked as ‘include’ and those that were agreed upon resolving disagreements. In the event of disagreement at this stage, a third reviewer (JJ) was involved, and a consensus was reached through discussion for final inclusion or exclusion. The selection process was documented using the PRISMA flow diagram (Fig. 1).

### Assessment of methodological quality

Two independent reviewers (DG and DA) critically appraised the methodological quality of the included studies. Due to the inclusion of all types of quantitative study designs, the methodological quality was assessed using a modified quality assessment tool for studies with diverse designs (QATSDD) [16]. The included studies were a mix of cross-sectional, case-control, cluster-randomised, quasi-experimental, and retrospective designs, and each study design was assessed using the tool. The tool was modified to remove two items specific to qualitative studies. The modified tool consists of 12 items designed to evaluate the methodological quality. For each of the remaining items, each was awarded a score of 0 – 3 (0=not at all, 1=very slightly, 2=moderate, and 3=complete). To get the methodological quality of the included studies, the scores were summed to generate the total score. Percentages were estimated for each study using their total score and categorised for ease of interpretation (>80 % as excellent, 50 %−80 % as good and <50 % as poor) (Supplementary Table S1) [16]. Any disagreement was addressed through discussion.

### Data extraction

DG extracted data from all included studies using a modified Joanna Briggs Institute (JBI) data extraction tool for prevalence review [17]. The extracted data included the author’s name and year of publication, population, setting, sample size, study design, type of data, outcome(s) measured, prevalence/proportion, mortality, disability, and cost of unintentional injuries from each study.

### Data analysis

Data were extracted using Microsoft Excel and imported into STATA version 18 for further cleaning, management and analysis. We descriptively summarised the results of the included studies on the prevalence, mortality, disability, and cost. We estimated the median and interquartile range for the prevalence and mortality. Prevalence was defined as the proportion of individuals who sustained the included unintentional injuries. Prevalence was estimated separately for studies that included only the injury population and those that included both the injured and non-injured populations (period prevalence). Mortality was the proportion of people who died among those who sustained the included unintentional injuries. A meta-analysis was conducted to estimate the pooled prevalence and mortality of unintentional injuries. Heterogeneity among studies was assessed to determine the most appropriate approach for pooling the results. This was done using Cochran’s Q and $I^{2}$ Statistic. When heterogeneity was statistically significant, a random-effect model was used to pool the prevalence. Additionally, subgroup analyses (by study design, age group (adult vs. children), sample size, and year of publication) were conducted to identify the sources of heterogeneity. Publication bias was assessed using the Egger test for small study effects.

## Results

### Study selection and characteristics

We identified 565 records from our electronic database search. After removing duplicates, 409 records were screened. We excluded 323 records through abstract and title screening and retrieved 86 full-text articles. Of the 86 articles reviewed in full, 46 met the inclusion criteria (Fig. 1).

The characteristics of the included studies are presented in Supplementary Table S2. Twenty-six studies (56.5 %) were registry-based (police and hospital registries), 18 (39.1 %) were population-based surveys (cross-sectional and cluster-randomised), and one (2.2 %) was a survey based in a hospital. Regarding the population involved in the studies, most of the studies (*n* = 31, 67.4 %) recruited only participants with injuries, while the remaining 15 (32.6%) recruited both injured and non-injured participants.

Five (10.9 %) studies reported on all the included unintentional injuries [[18], [19], [20], [21], [22]], and 22 (47.8 %) studies reported a single unintentional injury type [[23], [24], [25], [26], [27], [28], [29], [30], [31], [32], [33], [34], [35], [36], [37], [38], [39], [40], [41], [42], [43], [44], [45]]. Seven studies reported on two types of unintentional injuries [[46], [47], [48], [49], [50], [51], [52]], four studies reported on three types [9,[53], [54], [55]] and eight studies reported on four types [[56], [57], [58], [59], [60], [61], [62], [63]] (Supplementary Table S2). Of the 22 (47.8 %) studies reporting a single unintentional injury type, seven (31.8 %) were on RTIs, twelve (54.5 %) were on burns, and two (9.1 %) were on falls.

For quality assessment, the QATSDD scores ranged from 52.8 % to 88.9 % (Supplementary Table S1). Fifteen studies were categorised as having an excellent methodological quality, scoring above 80 %. The remaining studies demonstrated good methodological quality, with none scoring below 50 %.

### Prevalence of unintentional injuries

The prevalence of unintentional injuries was reported in twenty-seven (58.7 %) of the included studies. Out of these, 12 studies reported the prevalence of unintentional injuries from only an injured population [9,22,[46], [47], [48],[51], [52], [53], [54],^56^,^60^,^64^] and 15 studies estimated the prevalence from a population comprising both injured and non-injured populations [[19], [20], [21],^36^,^40^,^41^,^49^,^50^,^55^,[57], [58], [59],[61], [62], [63]], and the prevalence reported was period prevalence, over a 12-month recall period. The overall median and pooled prevalence of unintentional injury were estimated from the 15 studies that recruited injured and non-injured participants. These studies were all population-based surveys (both national and sub-national). The period prevalence of unintentional injuries in these studies ranged from 0.9 % to 57.9 %, with a median prevalence of 20.5 % and an IQR of 12.7 % (11.7 %, 24.4 %).

**Fig. 2:**
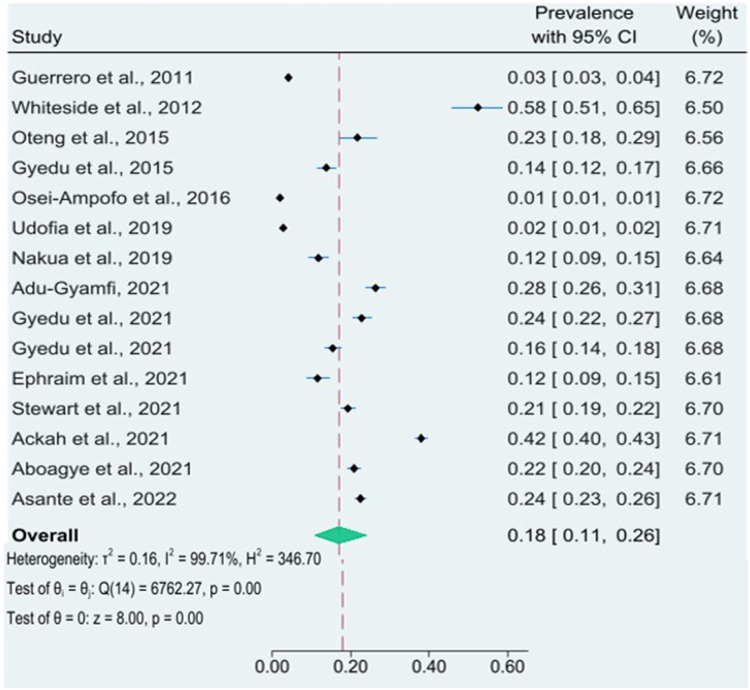
Pooled prevalence of unintentional injuries in Ghana.

The pooled period prevalence of unintentional injuries from these studies was 18 % (95 % CI: 11 % to 26 %) with a high level of heterogeneity across studies ($I^{2}$=99.71 %, *p* < 0.001) (Fig. 2). Thus, a prevalence rate of 180 unintentional injuries per 1000 person-year (95 % CI: 110 to 260 unintentional injuries/1000 person-year). When we stratified by adults and children, the pooled prevalence of unintentional injuries among adults was 11 % (95 % CI: 3 % to 22 %), and 23 % (95 % CI: 14 % to 34 %) among children (Supplementary Figure 1).

Subgroup analysis was performed since the analysis showed a high level of heterogeneity. The analysis was conducted using the study design, sample size, age group, and year of publication. The results showed a slight reduction in heterogeneity among studies conducted using cluster-randomised surveys ($I^{2}$ = 91 %, *p* < 0.001). The analysis by sample size also indicated a slight reduction in heterogeneity among studies that used sample sizes of less than 500 ($I^{2}$=90 %, *p* < 0.001). *Publication bias*: The Egger test for small study effects revealed no evidence of publication bias (*p* = 0.104). The prevalence of unintentional injuries from studies using data from only injured populations (*n* = 12) is summarised in Supplementary Table 3.

### Mortality

Twenty-two (47.9 %) of the studies reported on mortality from the specified unintentional injuries in this study [9,[23], [24], [25],[27], [28], [29], [30], [31], [32], [33], [34], [35],^38^,^39^,[42], [43], [44],^47^,^60^,^64^,^65^]. All these studies were based on national registries, which included medical records and road crash databases, as survey studies lacked mortality data. To estimate the pooled mortality from unintentional injuries, studies that exclusively reported mortality data, such as those based solely on road crash databases, were excluded [60,64,65]. These studies lacked a denominator population, resulting in a mortality proportion of one (1), which would have artificially inflated the pooled estimate and distorted the pooled proportion of mortality burden. Therefore, the median and pooled mortality from unintentional injuries were estimated from 19 studies [9,[23], [24], [25],[27], [28], [29], [30], [31], [32], [33], [34], [35],^38^,^39^,[42], [43], [44],^47^]. The mortality from unintentional injuries reported in these studies ranged between 0.9 % and 67.7 %. The estimated median mortality was 15.7 %, with an IQR of 13.8 %.

**Fig. 3:**
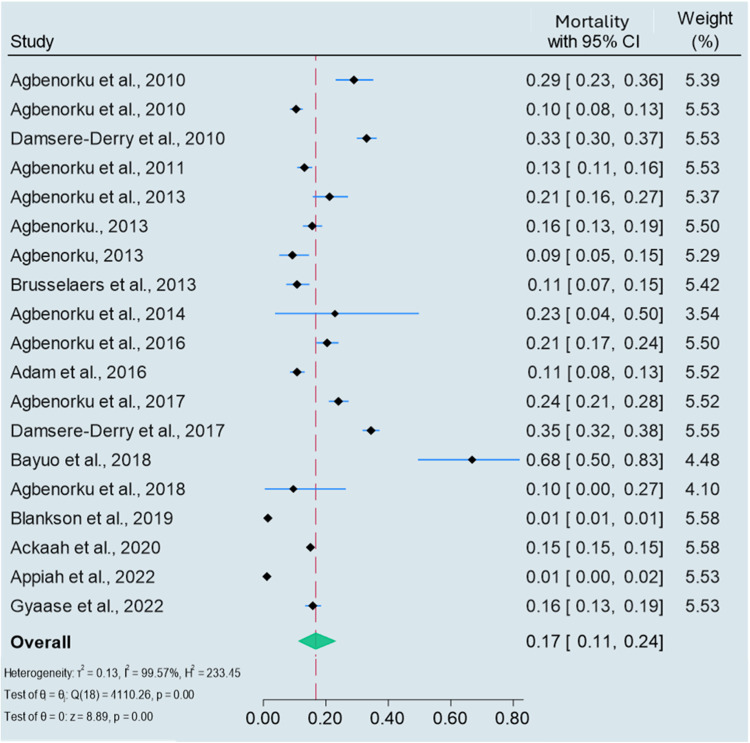
Pooled mortality from unintentional injuries in Ghana.

The pooled mortality from unintentional injuries in these studies was 17 % (95 % CI: 11 % to 24 %), with a high level of heterogeneity across studies ($I^{2}$ = 99.57 %, *p* < 0.001) (Fig. 3). Thus, 170 deaths per 1000 unintentional injury-year (95 % CI: 110 to 240 deaths per 1000 unintentional injury-year). Due to high heterogeneity, a subgroup analysis was conducted using sample size and year of publication. Heterogeneity was marginally reduced for studies with a sample size below 500 ($I^{2}$ = 95.57 %, *p* < 0.001). The analysis by year of publication revealed a slight reduction in heterogeneity among studies conducted before 2015 ($I^{2}$ = 94.92 %, *p* < 0.001).

*Publication bias:* The Egger test for small study effects revealed no evidence of publication bias (*p* = 0.804).

### Prevalence and mortality by unintentional injury mechanism

Table 1 presents a summary of prevalence and mortality among individuals who sustained an unintentional injury, categorised by injury mechanism. Among the specific types of unintentional injuries, RTIs had the highest median prevalence (57.8 %), followed by falls at 35.9 %. Drowning recorded the lowest median prevalence at 1.6 %

**Table 1:** Summary of specific unintentional injuries: prevalence and mortality estimates.

Injury mechanism	Prevalence		Mortality	
	Range (%)	Median (%)	Range (%)	Median (%)
RTIs	3.2 to 94.4	57.8	0.9 to 81.6	42.0
Falls	3.4 to 96.6	35.9	1.7 to 18.3	14.7
Burns	0.9 to 76.8	9.2	4.2 to 67.7	14.4
Drowning	0.2 to 2.6	1.6	9.1 to 47.9	20.7
Poisoning	1.0 to 25.2	2.8	2.0 to 5.9	3.7

For mortality, RTIs had the highest median mortality (42.0 %), followed by drowning (20.7 %). And the lowest was poisoning (3.7 %).

### Cost of treating unintentional injuries

Two studies from hospitals reported the medical costs associated with managing unintentional injuries. One study determined the cost of treating all injuries [53], while the other focused on the cost of treating only burn patients [24]. The associated cost for treating unintentional injuries reported in these studies was extracted and presented. Agbenorku et al [24] reported the average cost of treating burns as US$1731 (individual level) and at the population level, the average annual direct cost of treating all burn patients at the Komfo Anoakye Teaching Hospital as US$56,230.00. The study by Blankson and colleagues [53] estimated the annual household cost of treating injuries to patients who sought treatment at the Korle-Bu Teaching Hospital. The annual cost of treating injuries included in this review was extracted and presented in this study. At the population level, the total annual cost (direct and indirect) from unintentional injuries in the study was reported as US$8841,536.50 (direct = US$7209,349.50 and indirect = US$1632,187.00). The associated cost of treating RTIs was US$6730,862.89, US$1645,736.50 for fall injuries, and US$464,937.11 for burn injuries. On the individual level, the average cost of treating RTIs was US$1687.65 (direct = US$1289.40 and indirect = US$398.25), fall was US$975.61 (direct = US$838.80 and indirect = US$136.81), and burn was US$1676.36 (direct = US$1,370.63 and indirect = US$305.73).

### Disability

Only one study reported on the disability associated with unintentional injuries. It reported on functional disability associated with burns at the Komfo Anoakye Teaching Hospital. Scar contractures and disfigurements were the primary forms of disability reported in the study. The reported disabilities resulted in activity limitations, such as impeded arm elevation, inability to fold the palm, inability to move the foot and toes, and inability to bend the knee [26] (Table 2).

**Table 2:** Individual and population-level direct and indirect costs to the injured person by mechanism.

	Injury	Cost type	Cost (US$)	
			*Individual level*	*Population level*
			*Average*	*Total/year*
Blankson et al [53]	RTIs	Direct	1289.40	5499,261.76
		Indirect	398.25	1231,601.13
		Total	1687.65	6730,862.89
	Falls	Direct	838.80	1319,544.98
		Indirect	136.81	326,191.52
		Total	975.61	1645,736.50
	Burns	Direct	1370.63	390,542.73
		Indirect	305.73	74,394.38
		Total	1676.36	464,937.11
				*Average/year*
Agbenorku et al [24]	Burn	Direct	1731	56,230.00

## Discussion

The findings of the study show a high prevalence, mortality, cost of management, and limited data on the economic burden and disabilities following unintentional injuries.

The pooled prevalence of unintentional injuries estimated in this study, 18 % (95 % CI: 11 %–26 %), is higher than that reported in the Global Burden of Disease (GBD) study over the years [66]. This discrepancy may stem from methodological differences. Unlike the GBD, which incorporates multiple data sources, including official records and vital statistics, this review was restricted to peer-reviewed scientific publications. However, it would be inappropriate to conclude that the GBD underestimated the prevalence in Ghana, as only a limited number of studies included in this review were nationally representative. Furthermore, those that were nationally representative focused exclusively on children and older adults. The remaining studies were conducted in urban settings, specifically in Accra and Kumasi, and did not account for regional differences across the country.

Compared to other LMICs, the prevalence reported in this review is higher than in countries such as Kenya (15.3 %) [67] and Bangladesh (3.4 %) [68]. We found a higher prevalence among children (23 %) compared to adults (11 %), which is nearly three times higher than that reported by Ruiz-Casares and colleagues [69] for Sub-Saharan Africa. Given the limited national representativeness of the included studies, the reported prevalence should not be interpreted as a reflection of the national situation. Notwithstanding, evidence suggests that rural areas contribute significantly to the burden of injuries [70], suggesting that the true prevalence may be equal to or even higher than what is reported here. The high burden of unintentional injuries demonstrated from this systematic review calls for attention and further research to identify the contributing factors and feasible interventions to minimise the burden in Ghana. Also, it is essential that the national annual health report include the incidence of injuries and stratify them by the intentionality of the injury.

The findings on mortality from unintentional injuries align with global trends, highlighting a high burden of deaths from such injuries. Despite not including all unintentional injuries in this review, we found a high burden of mortality. It is well established that unintentional injuries account for over 72 % of all injury-related deaths [1]. As an LMIC, the high mortality found in this study supports the existing evidence that unintentional injuries pose a substantial burden across LMICs [1]. The finding highlights the need for targeted interventions and policy changes to enhance safety and reduce preventable deaths.

The major contributor to the high prevalence and mortality was RTIs. This is not surprising because, across the globe, RTIs have been identified as the leading cause of unintentional injuries [71,72]. Among all injury-related deaths, RTIs account for over 24 %, a proportion nearly equivalent to the combined mortality from self-harm and interpersonal violence [72]. The finding highlights the significant role RTIs play in contributing to injury-related mortality and the need for a comprehensive public health response in Ghana. Over the years, interventions and strategies were made to address this challenge, such as the UN Decade of Action for Road Safety (2011–2020 and 2021–2030) [73,74] and the Ghana National Road Safety Strategies I-III. Given the already substantial burden of RTIs and the additional insights provided by this study, prioritising road safety interventions is imperative. While both global and national mandates exist, there must be a renewed and focused commitment to achieving the Decade of Action for Road Safety target of reducing road traffic fatalities by 50 % by 2030. Globally, the evidence base for effective interventions is well-established, ranging from the enforcement of traffic laws (including helmet use, seatbelt compliance, and speed regulation) to improvements in road infrastructure, vehicle safety standards, public education on road use, and strengthened emergency medical response systems [75] Despite this, implementation in Ghana remains weak across these domains. Realising the ambitions of the second UN Decade of Action for Road Safety (2021–2030) hinges on countries like Ghana elevating road safety as a national priority and systematically adopting proven, evidence-based strategies.

Drowning was also noted as a significant contributor to the high mortality following unintentional injuries in Ghana. Historically, drowning has not received much attention in the country, potentially due to cultural beliefs surrounding water bodies. In most parts of Ghana, water bodies are considered sacred, and drowning is viewed as divine retribution in these places [76]. Despite the supernatural attachment, drowning has been a growing concern, with at least 1360 deaths annually [77]. The findings highlight the need for national dialogue and targeted strategies, including improved urban planning and community awareness programs to prevent drowning-related incidents, which are currently underrepresented in public health discourse. National-level campaigns and collaboration with local governments can promote safer environments, especially in areas near water bodies.

Similar to a study from Vietnam [78], our review found that the cost of treating unintentional injuries, particularly RTIs and burns, was substantial. The average cost of treating RTIs and burns was US$1688 and US$1676 per individual, respectively [53]. This amount is particularly burdensome for the injured and their families, considering the evidence that most injuries occur among people of low economic status [[79], [80], [81]]. The burden is further compounded by the fact that, in Ghana, the average annual per capita income is significantly lower (US$1133 in urban areas and US$407 in rural areas) than the treatment costs [82]. Although the cost we found was high, the review highlights the lack of research on the economic burden of unintentional injuries, underscoring the need for further studies in this area. The high cost of treating unintentional injuries, such as RTIs and burns, highlights the need for government intervention. Expanding access to the country’s National Health Insurance Scheme to treat and increase financial support for the injured and their families through insurance or social welfare programs could ease the economic burden on individuals and their families.

This study revealed a paucity of evidence on the long-term outcomes of unintentional injuries, with only one study addressing functional disabilities resulting from burns [26]. This study reported scar contractures and disfigurements, both of which have profound implications for activities of daily living and overall quality of life. The lack of research on disability following unintentional injuries highlights a critical gap in the literature. Research studies are necessary to document the prevalence and types of disabilities resulting from various forms of unintentional injuries to inform rehabilitation practices, health insurance, and policy development for long-term disabilities.

## Conclusion

This review highlights the heavy burden of unintentional injuries in Ghana, with high prevalence and mortality, especially from RTIs and drowning. Addressing this requires targeted interventions such as road safety and drowning prevention. Data on economic impacts are limited, but treatment costs are significant. Critical research gaps include the economic burden and long-term disability outcomes. Future studies should focus on these to guide policy and resource allocation. Multisectoral collaboration among public health, policymakers, researchers, and organisations is essential to reduce these injuries.

## Funding

DG is supported by the University International Postgraduate Award (UIPA) from the University of New South Wales (UNSW). JJ is supported by the National Health and Medical Research Council (NHMRC) as an emerging leader 1.

## Dissemination of results

The results of this systematic review and meta-analysis have been presented to some stakeholders in Ghana. An abstract of the review was submitted for the Cochrane Africa Indaba 2025. We also planned to present these results during the 2026 World Conference on Injury Prevention and Safety Promotion (Safety 2026) in South Africa.

## CRediT authorship contribution statement

**Daniel Gyaase:** Conceptualization, Methodology, Data curation, Formal analysis, Writing – review & editing. **Deepti Beri:** Data curation, Validation, Formal analysis, Writing – review & editing. **Stacie Powell:** Validation, Writing – review & editing. **Nipuna Cooray:** Validation, Writing – review & editing. **Margie Peden:** Supervision, Writing – review & editing. **Julie Brown:** Supervision, Writing – review & editing. **Jagnoor Jagnoor:** Conceptualization, Methodology, Supervision, Writing – original draft, Writing – review & editing.

## Declaration of competing interest

The authors declare no conflict of interest

## Appendix Supplementary materials

Image, application 1
