# Supplementary material for: Examining the burden of unintentional injuries in Ghana: A systematic review and meta-analysis
Source: Afr J Emerg Med. 2025 Sep 18;15(4):100907. doi: 10.1016/j.afjem.2025.100907 (PMC12481059; doi:10.1016/j.afjem.2025.100907)
Supplement: Supplementary file 1 [file mmc1.docx]

Supplementary Table S1: Quality assessment of included studies using the Quality Assessment Tool for Studies with Diverse Designs (QATSDD)

| Included studies | QATSDD Criteria | | | | | | | | | | | |  |  |
| --- | --- | --- | --- | --- | --- | --- | --- | --- | --- | --- | --- | --- | --- | --- |
|  | **1** | **2** | **3** | **4** | **5** | **6** | **7** | **8** | **9** | **10** | **11** | **12** | **Score** | **%score** |
| Agbenorku et al 2010 | 0 | 0 | 3 | 3 | 3 | 3 | 3 | 3 | 0 | 0 | 1 | 0 | 19 | 52.8 |
| Brusselaers et al 2013 | 1 | 3 | 3 | 3 | 3 | 3 | 3 | 3 | 3 | 3 | 3 | 0 | 31 | 86.1 |
| Agbenorku et al 2010 | 0 | 3 | 3 | 3 | 3 | 3 | 1 | 3 | 3 | 3 | 2 | 0 | 27 | 75.0 |
| Agbenorku et al 2018 | 0 | 3 | 3 | 3 | 3 | 3 | 1 | 3 | 3 | 3 | 2 | 1 | 28 | 77.8 |
| Agbenorku 2013 | 0 | 1 | 3 | 3 | 3 | 3 | 1 | 3 | 3 | 1 | 1 | 0 | 22 | 61.1 |
| Torgbenu et al 2017 | 0 | 3 | 3 | 3 | 3 | 3 | 3 | 3 | 3 | 3 | 3 | 0 | 30 | 83.3 |
| Akakpo et al 2020 | 0 | 1 | 3 | 3 | 3 | 1 | 2 | 3 | 3 | 3 | 2 | 0 | 24 | 66.7 |
| Stewart et al 2021 | 0 | 0 | 3 | 3 | 3 | 3 | 3 | 3 | 3 | 3 | 3 | 0 | 27 | 75.0 |
| Adu-Gyamfi 2020 | 0 | 3 | 3 | 3 | 3 | 2 | 3 | 3 | 3 | 3 | 3 | 0 | 29 | 80.6 |
| Agbenorku et al 2016 | 0 | 3 | 3 | 3 | 3 | 1 | 2 | 3 | 3 | 3 | 3 | 1 | 28 | 77.8 |
| Agbenorku 2013 | 0 | 3 | 3 | 3 | 3 | 1 | 1 | 3 | 3 | 3 | 1 | 0 | 24 | 66.7 |
| Appiah et al 2023 | 0 | 2 | 2 | 3 | 3 | 3 | 3 | 3 | 3 | 3 | 3 | 0 | 28 | 77.8 |
| Gyaase et al 2023 | 0 | 3 | 3 | 0 | 3 | 1 | 1 | 3 | 3 | 3 | 3 | 0 | 23 | 63.9 |
| Agbenorku et al 2014 | 0 | 0 | 3 | 3 | 3 | 3 | 1 | 3 | 3 | 3 | 1 | 2 | 25 | 69.4 |
| Forson et al 2020 | 0 | 3 | 3 | 3 | 3 | 3 | 2 | 3 | 3 | 3 | 2 | 3 | 31 | 86.1 |
| Agbenorku et al 2017 | 0 | 3 | 3 | 3 | 3 | 1 | 1 | 2 | 3 | 3 | 3 | 0 | 25 | 69.4 |
| Agbenorku et al 2011 | 0 | 3 | 3 | 3 | 3 | 3 | 3 | 3 | 3 | 3 | 2 | 2 | 31 | 86.1 |
| Bayuo et al 2018 | 0 | 1 | 3 | 1 | 3 | 3 | 3 | 3 | 3 | 3 | 2 | 0 | 25 | 69.4 |
| Buunaaim et al 2023 | 0 | 3 | 3 | 3 | 3 | 1 | 3 | 2 | 3 | 3 | 2 | 0 | 26 | 72.2 |
| Blankson et al 2019 | 0 | 2 | 3 | 3 | 3 | 3 | 3 | 3 | 3 | 3 | 2 | 0 | 28 | 77.8 |
| Ackah et al 2021 | 0 | 2 | 3 | 3 | 3 | 3 | 3 | 1 | 3 | 3 | 3 | 0 | 27 | 75.0 |
| Baiden et al 2022 | 0 | 2 | 3 | 3 | 3 | 3 | 3 | 3 | 3 | 3 | 3 | 3 | 32 | 88.9 |
| Frimpong et al 2021 | 0 | 2 | 3 | 3 | 3 | 3 | 0 | 2 | 3 | 3 | 1 | 0 | 23 | 63.9 |
| Gyedu et al 2021 | 0 | 3 | 3 | 3 | 3 | 3 | 3 | 3 | 3 | 3 | 3 | 0 | 30 | 83.3 |
| Adam et al 2016 | 0 | 3 | 3 | 3 | 3 | 3 | 3 | 3 | 3 | 3 | 3 | 0 | 30 | 83.3 |
| Gyedu et al 2014 | 0 | 3 | 3 | 3 | 3 | 3 | 3 | 3 | 3 | 3 | 3 | 0 | 30 | 83.3 |
| Oteng et al 2015 | 0 | 3 | 3 | 1 | 3 | 3 | 1 | 3 | 2 | 2 | 1 | 0 | 22 | 61.1 |
| Blankson et al, 2020 | 0 | 2 | 3 | 3 | 3 | 3 | 1 | 3 | 3 | 3 | 3 | 0 | 27 | 75.0 |
| Osei-Ampofo et al 2016 | 0 | 3 | 1 | 3 | 3 | 0 | 3 | 3 | 3 | 2 | 3 | 0 | 24 | 66.7 |
| Nakua et al 2019 | 0 | 3 | 3 | 3 | 3 | 3 | 3 | 3 | 3 | 3 | 3 | 0 | 30 | 83.3 |
| Ohene et al 2010 | 0 | 3 | 3 | 3 | 3 | 3 | 3 | 3 | 3 | 3 | 3 | 0 | 30 | 83.3 |
| Agbenorku 2013 | 0 | 3 | 3 | 3 | 3 | 3 | 3 | 3 | 3 | 3 | 1 | 0 | 28 | 77.8 |
| Whiteside et al. 2012 | 0 | 3 | 3 | 3 | 3 | 3 | 1 | 3 | 3 | 3 | 1 | 0 | 26 | 72.2 |
| Guerrero et al 2011 | 0 | 3 | 3 | 3 | 2 | 3 | 3 | 3 | 3 | 3 | 3 | 0 | 29 | 80.6 |
| Damsere-Derry et al 2010 | 0 | 2 | 2 | 3 | 3 | 1 | 1 | 2 | 3 | 3 | 3 | 0 | 23 | 63.9 |
| Agbenorku et al 2013 | 0 | 2 | 3 | 3 | 3 | 3 | 1 | 3 | 3 | 3 | 3 | 0 | 27 | 75.0 |
| Aboagye et al 2021 | 0 | 3 | 3 | 3 | 3 | 3 | 2 | 3 | 3 | 3 | 3 | 3 | 32 | 88.9 |
| Ephraim et al. 2021 | 0 | 3 | 3 | 3 | 3 | 3 | 1 | 3 | 3 | 3 | 3 | 0 | 28 | 77.8 |
| Udofia et al 2019 | 0 | 2 | 3 | 3 | 3 | 3 | 2 | 3 | 3 | 3 | 3 | 0 | 28 | 77.8 |
| Ossei et al 2019 | 0 | 2 | 3 | 3 | 3 | 2 | 2 | 3 | 3 | 3 | 1 | 0 | 25 | 69.4 |
| Damsere-Derry et al 2017 | 0 | 3 | 3 | 3 | 3 | 3 | 3 | 3 | 3 | 3 | 3 | 2 | 32 | 88.9 |
| Ackaah et al 2020 | 0 | 1 | 2 | 2 | 3 | 1 | 1 | 2 | 3 | 3 | 2 | 0 | 20 | 55.6 |
| Asante et al 2022 | 0 | 2 | 3 | 3 | 3 | 3 | 3 | 3 | 3 | 3 | 3 | 3 | 32 | 88.9 |
| Ametefe et al 2016 | 0 | 3 | 2 | 3 | 3 | 1 | 1 | 3 | 3 | 3 | 1 | 0 | 23 | 63.9 |
| Gyedu et al 2021 | 0 | 2 | 3 | 3 | 3 | 3 | 3 | 3 | 3 | 3 | 3 | 0 | 29 | 80.6 |

**QATSDD Criteria**: (1) Theoretical framework; (2) Aims/objectives; (3) Clear description of study setting; (4) Evidence of sample size considered; (5) Representative sample of target group; (6) Description of procedure for data collection; (7) Rationale for choice of data collection tool(s); (8) Detailed recruitment data; (9) Fit between research question and method of data collection (Quantitative only); (10) Fit between research question and method of analysis (Quantitative only); (11) Good justification for analytical method selected; (12) Strengths and limitations.

**QATSDD rating scale**: 0 = not at all; 1 = very slightly; 2 = moderately; 3 = complete.

Supplementary Table S2: Characteristics of included studies

| Authors | Year | Study design | Study type | Data type | Participants | Sample size | Outcome measured | Types of injuries reported |
| --- | --- | --- | --- | --- | --- | --- | --- | --- |
| Agbenorku et al. | 2010 | Retrospective | Registry-based | Secondary | Injured | 212 | Injury and Death | Burns |
| Ohene et al. | 2010 | Retrospective | Registry-based | Secondary | Injured | 882 | Death | RTIs, burns, drowning and poison |
| Agbenorku et al. | 2010 | Prospective | Registry-based | Secondary | Injured | 826 | Injury and Death | Burns |
| Damsere-Derry et al. | 2010 | Retrospective | Registry-based | Secondary | Injured | 812 | Injury and Death | RTIs |
| Agbenorku et al. | 2011 | Retrospective | Registry-based | Secondary | Injured | 731 | Injury and Death | Burns |
| Agbenorku et al. | 2013 | Retrospective | Registry-based | Secondary | Injured | 197 | Injury and Death | Burns |
| Agbenorku | 2013 | Cross-sectional | Survey | Primary | Injured | 70 | Disability | Burns |
| Agbenorku | 2013 | Retrospective | Registry-based | Secondary | Injured | 511 | Injury and Death | Burns |
| Agbenorku | 2013 | Retrospective | Registry-based | Secondary | Injured | 141 | Injury and Death | Burns |
| Brusselaers et al. | 2013 | Retrospective | Registry-based | Secondary | Injured | 261 | Injury and Death | Burns |
| Agbenorku et al. | 2014 | Retrospective | Registry-based | Secondary | Injured | 13 | Injury and Death | Burns |
| Agbenorku et al. | 2016 | Retrospective | Registry-based | Secondary | Injured | 487 | Injury and Death | Burns |
| Ametefe et al. | 2016 | Retrospective | Registry-based | Secondary | Injured | 185 | Injury | RTIs and falls |
| Adam et al. | 2016 | Retrospective | Registry-based | Secondary | Injured | 671 | Injury and Death | RTIs and falls |
| Agbenorku et al. | 2017 | Retrospective | Registry-based | Secondary | Injured | 681 | Injury and Death | Burns |
| Torgbenu et al. | 2017 | Cross-sectional | Survey | Primary | Injured | 269 | Injury | RTIs and falls |
| Damsere-Derry et al. | 2017 | Retrospective | Registry-based | Secondary | Injured | 1149 | Injury and Death | RTIs |
| Bayuo et al. | 2018 | Retrospective | Registry-based | Secondary | Injured | 31 | Injury and Death | Burns |
| Agbenorku et al. | 2018 | Retrospective | Registry-based | Secondary | Injured | 21 | Injury and Death | Burns |
| Ossei et al. | 2019 | Retrospective | Registry-based | Secondary | Injured | 1470 | Death | RTIs, falls, drowning, and poison |
| Blankson et al. | 2019 | Retrospective | Registry-based | Secondary | Injured | 17860 | Injury and Death | RTIs, falls and burns |
| Ackaah et al. | 2020 | Retrospective | Registry-based | Secondary | Injured | 60272 | Injury and Death | RTIs |
| Blankson et al. | 2020 | Cross-sectional | Survey | Primary | Injured | 301 | Cost of injury | RTIs, falls and burns |
| Forson et al. | 2020 | Cross-sectional | Survey | Primary | Injured | 171 | Injury | RTIs, falls and burns |
| Akakpo et al. | 2020 | Retrospective | Registry-based | Secondary | Injured | 1187 | Death | RTIs, falls, burns, drowning and poison |
| Appiah et al. | 2022 | Case-control | Hospital-based | Primary | Injured | 759 | Injury and Death | RTIs |
| Baiden et al. | 2022 | Retrospective | Registry-based | Secondary | Injured | 6314 | Injury | RTIs, falls, burns, drowning and poison |
| Frimpong et al. | 2021 | Retrospective | Registry-based | Secondary | Injured | 268 | Injury | RTIs and falls |
| Buunaaim et al. | 2022 | Retrospective | Registry-based | Secondary | Injured | 69 | Injury | RTIs and falls |
| Gyaase et al. | 2023 | Quasi-experimental | Registry-based | Secondary | Injured | 769 | Injury and Death | RTIs |
| Guerrero et al. | 2011 | Cross-sectional | Survey | Primary | Injured & non-injured | 5128 | Injury | RTIs |
| Whiteside et al. | 2012 | Cross-sectional | Survey | Primary | Injured & non-injured | 176 | Injury | RTIs, falls, burns, and poison |
| Oteng et al. | 2015 | Cross-sectional | Survey | Primary | Injured & non-injured | 254 | Injury | RTIs, falls, burns, and poison |
| Gyedu et al. | 2015 | Cross-sectional | Survey | Primary | Injured & non-injured | 637 | Injury | RTIs, falls, burns, drowning and poison |
| Adu-Gyamfi | 2021 | Cross-sectional | Survey | Primary | Injured & non-injured | 957 | Injury | Falls and burns |
| Gyedu et al. | 2021 | Cluster-randomized | Survey | Primary | Injured & non-injured | 1016 | Injury | RTIs, falls, burns, drowning and poison |
| Gyedu et al. | 2021 | Cluster-randomized | Survey | Primary | Injured & non-injured | 1016 | Injury | Burns |
| Ephraim et al. | 2021 | Cross-sectional | Survey | Primary | Injured & non-injured | 358 | Injury | RTIs, falls and burns |
| Osei-Ampofo et al. | 2016 | Retrospective | Survey | Primary | Injured & non-injured | 11764 | Injury and Death | RTIs, falls, burns, and poison |
| Stewart et al. | 2021 | Cluster-randomized | Survey | Primary | Injured & non-injured | 1653 | Injury | RTIs, falls, burns, drowning and poison |
| Ackah et al. | 2021 | Cross-sectional | Survey | Primary | Injured & non-injured | 3592 | Injury | RTIs, falls, burns and poison |
| Aboagye et al. | 2021 | Cross-sectional | Survey | Primary | Injured & non-injured | 2058 | Injury | RTIs, falls, burns and poison |
| Udofia et al. | 2019 | Cross-sectional | Survey | Primary | Injured & non-injured | 3530 | Injury | Falls |
| Nakua et al. | 2019 | Cross-sectional | Survey | Primary | Injured & non-injured | 494 | Injury | Falls and burns |
| Asante et al. | 2022 | Cross-sectional | Survey | Primary | Injured & non-injured | 3227 | Injury | RTIs, falls, burns and poison |


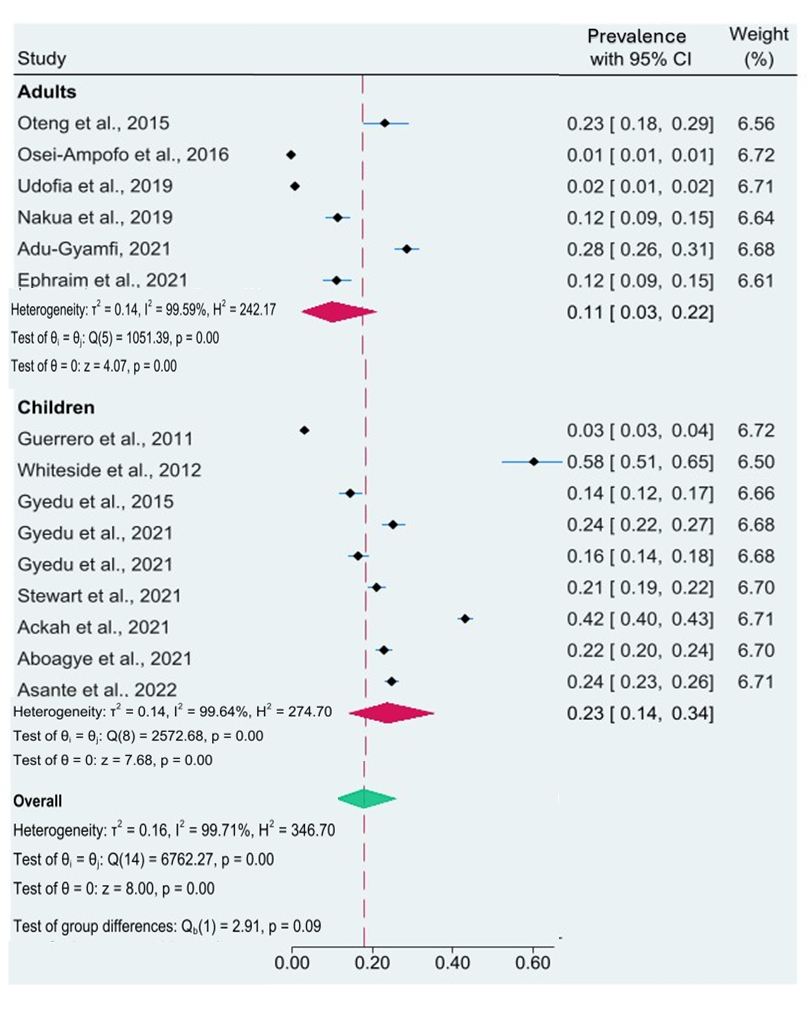


**Supplementary Figure S1**: Pooled prevalence of unintentional injuries by age group (adults and children)

For studies that recruited only injured participants (12 studies), the prevalence of unintentional injuries ranges from 13.5% to 94.6%, with a median prevalence of 67.0% and an IQR of 25.8%.

Specifically, the prevalence of RTIs among reported unintentional injuries ranged from 59.8% to 94.4%, with a median prevalence of 74.1%. Falls ranged from 3.4% to 37.6% with a median prevalence of 25.9%, and burns ranged from 0.9% to 12.9% with a median of 5.5%. The median prevalence of drowning poisoning was 0.2% and 2.3% respectively.

Supplementary Table 3: Prevalence of unintentional injuries from only injured population

|  | **Prevalence** | |
| --- | --- | --- |
|  | **Range** | **Median** |
| Prevalence of unintentional injuries | 13.5% to 94.6% | 67.0% |
| **Specific injuries** |  |  |
| RTIs | 59.8% to 94.4% | 74.1% |
| Falls | 3.4% to 37.6% | 25.9% |
| Burns | 0.9% to 12.9% | 5.5% |
| Drowning | 0.2% to 0.2% | 0.2% |
| Poisoning | 2.3% to 2.3% | 2.3% |
